# Biophysical model of the role of actin remodeling on dendritic spine morphology

**DOI:** 10.1371/journal.pone.0170113

**Published:** 2017-02-03

**Authors:** C. A. Miermans, R. P. T. Kusters, C. C. Hoogenraad, C. Storm

**Affiliations:** 1 Theory of Polymers and Soft Matter, Department of Applied Physics, Eindhoven University of Technology, Eindhoven, The Netherlands; 2 Cell Biology, Faculty of Science, Utrecht University, Utrecht, The Netherlands; 3 Institute for Complex Molecular Systems, Eindhoven University of Technology, Eindhoven, The Netherlands; Universita degli Studi di Torino, ITALY

## Abstract

Dendritic spines are small membranous structures that protrude from the neuronal dendrite. Each spine contains a synaptic contact site that may connect its parent dendrite to the axons of neighboring neurons. Dendritic spines are markedly distinct in shape and size, and certain types of stimulation prompt spines to evolve, in fairly predictable fashion, from thin nascent morphologies to the mushroom-like shapes associated with mature spines. It is well established that the remodeling of spines is strongly dependent upon the actin cytoskeleton inside the spine. A general framework that details the precise role of actin in directing the transitions between the various spine shapes is lacking. We address this issue, and present a quantitative, model-based scenario for spine plasticity validated using realistic and physiologically relevant parameters. Our model points to a crucial role for the actin cytoskeleton. In the early stages of spine formation, the interplay between the elastic properties of the spine membrane and the protrusive forces generated in the actin cytoskeleton propels the incipient spine. In the maturation stage, actin remodeling in the form of the combined dynamics of branched and bundled actin is required to form mature, mushroom-like spines. Importantly, our model shows that constricting the spine-neck aids in the stabilization of mature spines, thus pointing to a role in stabilization and maintenance for additional factors such as ring-like F-actin structures. Taken together, our model provides unique insights into the fundamental role of actin remodeling and polymerization forces during spine formation and maturation.

## Introduction

A single neuron can contain hundreds to thousands of dendritic spines, actin-rich, micron-sized protrusions which project from dendritic shafts [1]. Mature spines consist of two basic compartments: a constricted region called the *neck*, supporting a bulbous *head* containing the postsynaptic site that makes contact with the axon of a nearby neuron. Spines come in a wide range of sizes and shapes, their lengths varying between 0.2 − 2*μm* and their volumes between 0.001 − 1*μm*^3^. Electron microscopy (EM) studies have identified several morphological categories of spines, such as thin, filopodium-like protrusions (‘thin spines’), and spines with a large bulbous head (‘mushroom spines’) [1–5]. Different live cell-imaging techniques have demonstrated that dendritic spines are highly dynamic structures, subject to constant morphological change even after birth.

**Fig 1:**
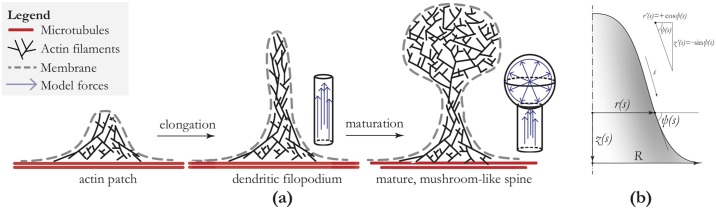
Outline of the model for spine formation and maturation. Panel (a): Cartoon of spine initiation, elongation and maturation. From left to right: ‘stubby spines’ dendritic filopodia or thin spines; mature, mushroom-like spines. In our mathematical model, we solve the shape equation based on the energy functional 1 (see S1 File, Eq (1)). In this study we show that, at least for the purposes of the force calculations, the results of the shape equation can be reproduced using the geometries that are also displayed in this figure. Panel (b): Definition of axisymmetric coordinate system that use for our models.

During neuronal development, dendrites initially appear as thin and hairlike filopodia (Fig 1). They are defined as having a length that is at least twice the width, and they do not display the bulbous head found on dendritic spines [5–7]. Filopodia are devoid of organelles and vesicles, and are composed primarily of actin filaments. These actin filaments are bundled and primarily aligned to the nascent spine. Filopodia are the precursors to dendritic spines, and their flexibility allows the establishment of synaptic contacts. Once the contact between a dendritic filopodium and a neighboring axon has been established, the spine-head begins to swell, taking on a more mushroom-like morphology. Over time, such recognizable mushroom spines become the prevalent structure on the dendritic shaft, and few filopodia remain.

The progressive shape change is neither random nor deterministic. Rather, it is thought to be correlated with the strength and maturity of each synapse [6, 7]. At the level of an individual spine, strengthening of a synapse is accompanied by modifications in the size of the spine. The prime mechanisms that drives structural plasticity is the modulation of actin dynamics in dendritic spine. Although the importance of actin remodeling as well as the synaptic signaling mechanisms involved in structural synaptic plasticity are well established [1, 5, 8], a general framework to correlate the state of the actin cytoskeleton to spine shape is lacking. Most importantly, it is not clear whether the actin is capable of autonomously *driving* the shape change, or whether the actin simply *follows* morphological transitions otherwise imposed.

Our model for spine dynamics uses the Canham-Helfrich formalism, an approach which has proven its strength in describing, both qualitatively and quantitatively, the deformation of biological membranes in numerous biological systems such as red-blood cells [9], membrane tethers [10] and binary or tertiary lipid mixtures in giant-unilamellar vesicles [11]. For a broad overview, we refer to [12] and many references therein. We analyze the interplay of the plasma membrane with the underlying actin cytoskeleton to quantify the forces that are required to prompt the initial formation of the spine, and its subsequent outward growth. We find that the forces generated by actin polymerization are sufficient for it to drive filopodium formation, and that the resulting dimensioning (quantified, for instance, by the ratio (protrusionwidth)/length) closely resembles those reported in experiments. A related theoretical model taking into account the interplay of the spine membrane with the actin cytoskeleton allows us, in addition, to compute the forces and energies required for spine head formation. It shows that the simultaneous presence of both branched actin filaments and bundled/aligned actin is required, and sufficient, to produce the typical mushroom-like spine morphology. Finally, our model also highlights the important role of additional physical processes in stabilizing the morphological features of mature spines. We discuss several candidate factors that may effect these processes, and conclude that these molecules are sufficiently rigid to be able to constrict the spine-neck to the extent reported in experiments. Our models do point to a fundamental role for actin remodeling in the process of spine formation and maturation. This finding supports earlier claims in the literature, and our model suggests novel experiments to further pin down the basic principles that control the structural plasticity of the brain.

## Materials and methods

Reflecting the approximate rotational symmetry of dendritic spines, we use an axisymmetric coordinate system consisting of an angle *ψ* with the horizontal, an arc-length parameter *s*, radial coordinate *r* and vertical coordinate *z*. Based on *in vivo* microscopy [1, 13, 14], we fix the angle of the shape at *ψ* = 0 on the edges of our integration interval *R*_base_ = 300 nm (cf. Table A in S1 File). This coordinate system is schematically displayed in Fig 1. The arc-length parameter s=0...S is used as the independent variable and *r*(*s*) and *ψ*(*s*) as the coordinates. This coordinate system fully determines the shape, and the vertical coordinate *z*(*s*) is recovered by the geometrical relation *z*′(*s*) = − sin *ψ*(*s*). The Canham-Helfrich energy functional that we use can be written [10, 11]

$$F=\frac{1}{2}K_{b}\int \text{d}a\;{\left(2H\right)}^{2}+\sigma \left(A-A_{0}\right)-f\left(L-L_{0}\right)-p_{\text{head}}V_{\text{head}},$$ (1)

where *K*_*b*_ ≈ 500pN · nm is the bending rigidity of the membrane [11], 2*H* = *ψ*′(*s*) + sin *ψ*(*s*)/*r*(*s*) is the mean curvature [15] (with *ψ*′(*s*) ≡ d*ψ*/d*s*), A=∫da is the surface area, *σ* is a surface tension which we use as a Lagrange multiplier to enforce the surface area, *f* is a point-force acting on the membrane, L=z(S)-z(0) is the height of the membrane, *p*_head_ is a pressure exerted on the membrane and $V_{\text{head}}$ is the volume of the spine-head. The first term in this energy functional—the one containing the mean curvature 2*H*—represents the bending energy of the membrane, which reflects the tendency of lipid bilayers to adopt a flat shape (or spherical in the case of vesicles with nonfixed volume). We use the surface tension *σ* and point-force *f* as Lagrange multipliers to enforce specific values of the surface-area $A_{0}$ and the height of the shape $L_{0}$ [16]. Within this paradigm, we interpret the surface-area, viz. amount of membrane available to the spine, as a quantity that encodes growth [17, 18]. The height of the shape reflects the cytoskeletal architecture of the spine, having a definite length. We stress that, due to the bending energy of the membrane, the point-force *f* acting on the membrane gives rise to a membrane deformation of a *finite size* [10, 19]. Thus, the singularity in the force-field does not translate into a singularity in the membrane shape. Moreover, there is experimental evidence that the filopodial force is strongly directional and orthogonal to the dendritic shaft [20], highlighting the importance of a point or point-like force in spine morphogenesis.

One of the principal goals of our work is to see whether the vertical forces *f* that are required—for producing the typical shapes observed in dendritic spines—can be generated by actin networks. Lastly, the pressure *p*_head_ models the force generated by branched actin networks in the spine-head, as we will discuss more thoroughly later in this paper. Although there is no obvious way of interpreting the Lagrange multiplier *σ*, the point-force *f* is simply the vertical part of the mechanical force that is exerted by the cytoskeleton on the spine membrane. We simplify the complicated and rich force generation of actin networks [5, 14, 21] to a vertical force (the point-force *f*) and a force normal to the membrane (the pressure *p*_head_) because we are interested in constructing a *minimal model* for spine formation. We will see that these two types of force—in combination with a mechanism for growth of the spines—suffice to describe basic aspects of dendritic spine morphogenesis.

Our choice to work at fixed total surface-area, rather than fixed surface tension, is inspired by two considerations: (i) Dendrites are finite in size, and the membrane that envelopes the dendritic shaft can only be as small as the underlying cytoskeleton of microtubules [1, 5]—therefore, we cannot regard the surroundings of the spine as a reservoir of freely accessible membrane. Instead, excess membrane needs to be transported, often by means of exocytic trafficking, in order to be available to the spine [17, 18]. (ii) On the dendritic shaft, generally, many spines exist side-by-side. In open boundary settings, such as those employed in [10], area is exchanged with a virtual bath outside the integration domain. In the dendritic shaft, however, no such bath exists as the next spine is likely also growing. Thus, a competition for membrane exists between proximate spines. For this reason, we choose to work with closed boundaries, prohibiting area to leak out of the domain of interest.

In our models, membrane does not leak away from the shape. We point out that there is biological evidence that cells strive to maintain their surface-tension [10, 22, 23]. Although this empirical fact might seem incompatible with our simulations, the ensembles of constant surface-area and constant surface-tension are—for the purpose of modeling mushroom-like spines—approximately equivalent. We elaborate on this in the supplementary information, but here we simply note that these ensembles quantitatively differ by only 5 − 40% (S1 File). Moreover, with regards to the findings that we present in the Conclusions, these two ensembles are interchangeable.

Using the Euler-Lagrange formalism, the energy functional Eq (1) can be transformed into a system of differential equations. These shape equations (S1 File, Eq (1)) have been numerically solved by means of a shooting-and-matching technique for a wide range of parameters $A_{0},L_{0}$ and several sets of boundary conditions (we drop the subscripts to $A_{0},L_{0}$ in the remainder of the paper). We ignore the stretching energy since lipid bilayer membranes can be regarded as approximately inextensible [24].

## Results and discussion

We use the Canham-Helfrich energy functional Eq (1) to model the growth of dendritic spine membranes. The growth sequence is schematically shown in Fig 1. We will show that this growth sequence can be explained qualitatively and quantitatively by simple models that incorporate the interaction of the actin cytoskeleton and the spine membrane. To that end, we will first determine how filopodia are formed by application of forces that the cytoskeleton exerts on the spine membrane. Then, we will show that the forces generated by a branched cytoskeleton, located at the top of the spine, will result in a bulbous head and a thin spine-neck. Finally, we will show that actin-membrane anchoring or ring-like molecules are another scenario for constraining a large head and long, thin neck. For the model calculations, we shall make repeated use of the physiologically relevant parameters that we have tabulated (see Table A in S1 File).

### Filopodium formation

**Fig 2:**
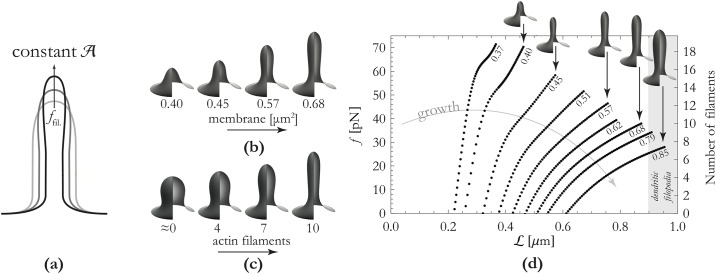
Outline of results for filopodium formation. Panel (a): Cartoon of qualitative effect of increasing force whilst the amount of membrane is kept constant. Panel (b): Effect of growth (viz. membrane addition) on filopodium morphology (if the force on the membrane is kept constant). These shapes experience a vertical force of 35pN corresponding to approximately 9 polymerizing actin filaments. Membrane addition results in substantial elongation of the filopodium. Panel (c): Effect of cytoskeletal remodelling (viz. actin polymerization) on filopodium morphology if the amount of membrane is kept constant. These shapes have a surface-area of 0.68*μ*m^2^. Increasing the number of polymerizing actin filaments leads to a marked change in morphology from a stubby-like morphology to a tubular shape. Panel (d): Force-extension curves of our models of dendritic filopodia for various values of the surface-area A. Numbers at curves indicate the surface-area in units of *μ*m^2^ whereby we used a radius of the base of the filopodia R_base_ = 300 nm.

It is well known that the actin cytoskeleton plays an important role in the formation of filopodia [1]. It has been hypothesized that polymerization of actin filaments and the resultant forces are sufficient for the formation of dendritic filopodia [14]. In order to theoretically investigate this possibility, we will present a model that includes extension of the actin cytoskeleton in growing filopodia. We note, that this analysis is not new—the same geometry is treated extensively in [25], for instance. It is instructive, however, to present here the results in a fixed area ensemble since this, we feel, more closely reflects the situation for a growing filopodium; the extension of the actin is likely faster than area addition. In this area-limited scenario, force-extension curves are markedly different from those in the constant surface tension ensemble; see Fig 2(d). We schematically display our modeling setup in Fig 2, and incorporate the quasistatic constraint presented by an actin bundle of a given length in the energy functional Eq (1) by fixing the height of the shape—thereby representing the vertical dimension of the cytoskeleton. This is congruent with single-molecule studies that show that actin polymerizes in a strongly directional fashion inside filopodia [20]. Moreover, based on *in vivo* microscopy [1, 13, 14], we fix the angle of the shape at *ψ* = 0 on the edges of our integration interval (at *r* = 300 nm, cf. Table A in S1 File). Growth of the cytoskeleton, or change in cytoskeletal architecture, is represented by incrementing this height constraint. We will first show that the forces that this rigid structure needs to exert on the spine membrane match the forces that are generated by actin polymerization. Then, we will show that the sequence of shapes as a consequence of polymerization of the actin cytoskeleton is similar to that of filopodium formation.

The protrusive forces that the rigid actin cytoskeleton exerts on the spine-membrane, will result in tube-like shapes, as can be seen in Fig 2. Also shown are the force-extension curves of these tubes for various values of the membrane surface-area. Since growth of dendritic spines and filopodia is mediated by exocytosis of endosomes at the synapse [17, 18], we can model the growth of spines by increasing the surface-area of the shape (also see the Methods part of this paper). Thus, we find that filopodia with more membrane require less force to be extended—in other words, membrane addition will result in further elongation of filopodia. Although the full force-extension relation shown in Fig 2 is non-trivial, the linear part for large extensions (i.e. large height L) can easily be understood from a theory that treats these structures as cylinders. From the bending energy of a cylinder (with given surface-area) $E=2\pi^{2}K_{b}L^{2}/A$ we find that the force f≡-∂E/∂L to extend this cylinder is linear in L. Applying this derivative, we find the force for producing filopodia $f\approx 4\pi^{2}K_{b}L/A$. This approximation turns out to be accurate to within 9% of the computed force-extension curves shown in Fig 2 (the error in this approximation decreases as the filopodium height increases). This is markedly different from the force required for pulling a tube from a reservoir (i.e. a (quasi-)infinite bath of membrane) of surface area. As is discussed in [10] (using detailed analytical and numerical calculations) the force for pulling a tube from such a reservoir converges to a constant for large extensions. If it were the case, then, that dendritic filopodia were connected to a bath of membrane, we would not expect dendritic filopodia to have a typical length. On the contrary, in that scenario dendritic filopodia would grow *ad infinitum* (given that the applied force is large enough to overcome an initial barrier). We assert that, within the paradigm of a conserved quantity of membrane available to the spine, a finite force will result in a definite length of the filopodia.

As can be seen in Fig 2, the force required for formation of dendritic filopodia is in the tens of piconewtons. The polymerization of actin is able to exert, on the average, a force of *f*_actin_ ≈ 3.8pN (from [26], see S1 File). We find, using the typical values for the length and surface-area (Table A in S1 File) and the aforementioned formula $f\approx 4\pi^{2}K_{b}L/A$, a minimal number of actin filaments of 5 − 15. Although we have not been able to find publications that mention the number of actin filaments in dendritic filopodia, examining EM of the cytoskeletal organization of dendritic filopodia from [5] suggests that filopodia typically have 6 − 10 filaments. This comparison tentatively verifies the plausibility of our model for filopodium formation. It is an empirical fact that actin filaments in the spine-neck are strongly directional [20], as our models show is necessary. This *oriented* or *aligned* actin organization may be due to actin factors, such as *profilin*, a protein that is known to localize at the spine-neck [27].

Our simulations span up to $A=0.85\;\mu m^{2}$ and L≈950nm. Following [28, 29], these shapes can be regarded as relatively small filopodia. Now, measuring the width of the corresponding tubular part of the shape, we find diameters in the order of 160 − 200nm. Indeed, [3] report values for the diameters of filopodia or thin spines in the range 90 − 210nm. Thus it is found that the simulations and experimental results have compatible ranges.

### The role of the actin cytoskeleton in spine maturation

As a consequence of synaptic activity, the spine volume may increase and there is a marked change in the qualitative morphology through formation of a bulbous spine-head [30, 31]. We have previously shown that simply adding membrane to dendritic filopodia results in larger filopodia, but not formation of a bulbous head. Therefore, an additional process is needed in order to produce mature spines. By which mechanisms does this qualitative change in morphology occur? In this part of the paper, we will show that the process of spine maturation can, at least in part, be ascribed to the interaction of the spine membrane with an isotropic actin meshwork.

As is the case for filopodia, it is known that the actin cytoskeleton is intimately linked to the size and shape of the spine head [1, 8], and therefore is essential to understanding spine maturation. By modeling the interaction of the cytoskeleton with the spine membrane, we will investigate the mechanical requirements for a volume increase and morphological transition (that is characteristic of spine maturation) to occur. We will show (by making use of the Canham-Helfrich energy Eq (1) and comparison with experiments) that branched actin filaments in the spine-head are plausibly responsible for the transition from dendritic filopodium to a mushroom-type morphology. Importantly, our models accurately predict various features of spine morphology and actin organization—despite the fact that these models lack many of the biological details. This shows that actin organization is one of the main drivers of spine maturation.

**Fig 3:**
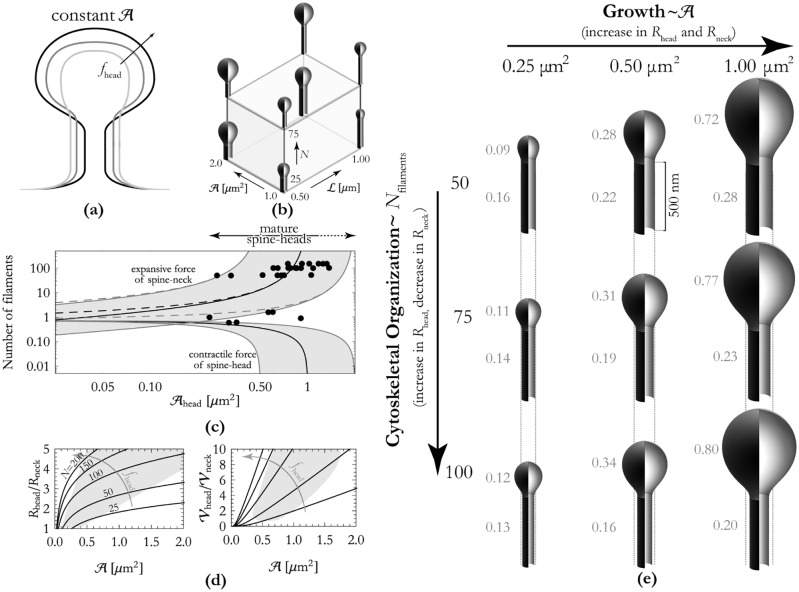
Outline of results for spine maturation. The spine bases have been left out in the renders in this figure. Panel (a): Cartoon showing qualitative effect of increasing force (viz. increasing the number of filaments in the spine-head) whilst the amount of membrane is kept constant. Increasing the number of actin filaments in the spine-head enlarges the spine-head and, at the same time, a thinning neck. Panel (b): Results of our model are combined in a three-dimensional growth-organization matrix, shown here with selected shapes. These shapes show clearly the effects of increasing the number of filaments in the head *N*, the total surface-area A and the length of the spine-neck L. Panel (c): The minimum number of actin filaments required in the cytoskeleton for sustaining the contractile force *f*_head_ that the spine-head membrane exerts and for counteracting the expansive force *f*_neck_ of the spine-neck. Band indicates typical values of the total amount of membrane $A=0.5\ldots 2.0\;\mu m^{2}$ (cf. Table A in S1 File). Dashed lines indicate number of actin filaments required for counteracting *f*_head_ + *f*_neck_. Empirical data (black circles) shows reasonable agreement with our model (data taken from from [32], S1 File). Panel (d): Ratio of head and neck radii (left) and volumes (right) for a number of actin filaments *N* = 25, 50, 100, 150, 200 (lower to upper curves). For these plots we used Eq 2 with $L_{\text{neck}}=500\;\text{nm}$. Experimental data from [13] is highlighted in gray. Panel (e): Effects of growth (membrane addition) and the number of actin filaments in the spine-head on spine morphology. In these models, we kept the total length of the spine-neck fixed. Dotted lines are a visual aid for showing how increasing the number of actin filaments in the spine-head results decreases the width of the spine-neck.

An outline of our model for spine maturation is displayed in Fig 3. We model the polymerization of actin in the spine-neck as a vertical force and polymerization in the spine-head as a radial force. The discrepancy between these two types of forces stems from the difference in cytoskeletal organization in spines—the spine-head predominantly contains branched actin whereas oriented or linear actin mainly localizes in the spine-neck [1, 5]. This gives rise to an approximately isotropic network of actin in the spine-head, contrary to the actin organization in spine-necks and dendritic filopodia [5]. The polymerization of these two manifestations of actin result respectively in a radial force and a directed force [20]. As is the case for our models for dendritic filopodia, we approximate the total surface-area of the spine as a constant since there is only a finite pool of membrane available on the dendrite (for a more detailed explanation, see the Methods part of this paper). This approximation, combined with the fact that lipid membranes are practically inextensible [24], leads to the following assertion: exerting an outward force on the spine-head results in the transportation of membrane from the spine-neck to the spine-head. More simply stated, cytoskeletal growth in the spine-head results in an increase of the size of the spine-head at the expense of a decrease in the neck width.

In order to make the above considerations quantitative, we propose a model for the spine membrane that is composed of a spine-neck connected to a spine-head. This combined system with a spine-neck and -head gives rise to an energy functional of the form Eq (1); applying the variational method to this energy functional leads to the shape equation reproduced in S1 File, Eq (1). Although it is possible to address these mature shapes in our variational framework, this involves dual integration domains, each with their own set of mechanical properties—thereby resulting in complicated matching conditions at the boundary. Tracking solution branches in the resulting two-stage shooting procedure proved quite intractable, and moreover is unnecessary when we recognize that the combination of a predominantly vertical force in the spine-neck, combined with the radial force in the spine-head drives the system towards a much simpler geometry (namely that of a cylinder for the spine-neck and a sphere for the spine-head). We will show that many of the interesting features of spine initiation and maturation can be captured by this simple model.

As displayed in Fig 3, we model *N* filaments in the spine-head that each apply an outward radial force *f*_actin_ = *f*_head_/*N* over a radius *R*_head_. The work performed by this force is *f*_head_ *R*_head_. Likewise, the energy required for attaining a neck of length $L_{\text{neck}}$ and radius *R*_neck_ is approximately $\pi K_{b}L_{\text{neck}}/R_{\text{neck}}$ (Eq (1)). The energy of the spine-head, modeled as a sphere, is the constant 8*πK*_*b*_ and hence does not enter the force balance. Balance of forces dictates that the total energy $\pi K_{b}L_{\text{neck}}/R_{\text{neck}}-f_{\text{head}}R_{\text{head}}$ is minimized. In order to insist conservation of membrane, we insert into the balance of forces the equation $A\approx 2\pi R_{\text{neck}}L_{\text{neck}}+A_{\text{head}}$ with A a constant. Taken together, this results in the following implicit equation that we can solve for $A_{\text{head}}$:

$$8\pi^{2}\sqrt{\pi }K_{b}{\left(\frac{L_{\text{neck}}}{A-A_{\text{head}}}\right)}^{2}-f_{\text{head}}/\sqrt{A_{\text{head}}}=0.$$ (2)

Given numerical values of *f*_head_, *K*_*b*_, A and $L_{\text{neck}}$, solving Eq 2 for $A_{\text{head}}$ returns all other geometrical quantities, e.g. *R*_neck_, *R*_head_ and $A_{\text{neck}}$. We have numerically solved this equation for a range of values for the total surface-area A and number of actin filaments in the spine-head *N* = *f*_head_/*f*_*actin*_. The influences of *growth*, encoded in the total surface-area of the spine A, and *cytoskeletal organization*, encoded in the number of actin filaments *N*, on spine morphology are combined in Fig 3(d). Using Eq 2 and solving for the radius of the spine-neck we find radii *R*_neck_ = 60 − 93nm (whereby we use estimates for the number of actin filaments *N* = 71 and the typical surface-areas $A=0.5-2.00\;\mu m^{2}$, cf. Table A in S1 File). This range agrees quite well with the experimentally observed ranges *R*_neck_ = 45 − 105nm by [3] and *R*_neck_ = 50 − 100nm by [13]. From the similarity of these ranges, we infer that at least a substantial part of the force that is exerted by the actin filaments in the spine-head is directed towards counteracting the expansive force of the spine-neck. Moreover, given numerical values of the total surface-area of the spine A we can solve for the number of actin filaments required for sustaining the spine-neck. We have done this for a wide range of surface-areas and reproduced the results in Fig 3(b). These computations show that a larger spine-head (with the same total quantity of membrane) requires more actin filaments to sustain it. This is in agreement with findings by [32] that show that the number of actin filaments increases substantially with increasing surface-area. In fact, the datapoints published in [32] match our model for $N(A_{\text{head}})$ (see Fig 3(c)). We have further used Eq 2 for computing the ratios or radii *R*_head_/*R*_neck_ and of volumes $V_{\text{head}}/V_{\text{neck}}$, shown in Fig 3(c). We have found that both the numerical values of *R*_head_/*R*_neck_ and the upward trend w.r.t. $A_{\text{head}}$ of this metric agree well with data published by [13] if we use for the number of actin filaments *N* ≈ 70 − 150. Thus, the renders shown in Fig 3(d) appear to be in the physiologically relevant regime. Moreover, this number for the actin filaments appears to be supported by empirical data that shows *N* ≈ 50 − 150 (we estimated this on the basis of data published in [32], see S1 File).

Importantly, we find that the minimum number of actin filaments in the spine-head for mushroom spines is an order of magnitude greater than the number of actin filaments required for filopodium initiation (compare Figs 3(d) and 2(d)). Additionally, the force exerted in the spine-neck is a vertical one, whereas the force in the spine-head is omnidirectional. This implies that a protein factor is required that branches the actin filaments in the spine-neck such as Arp2/3, a protein that is known to localize to the spine-head [5].

### Relationship between actin-membrane anchoring and ring-like complexes on spine morphology

We have discussed possible links between the cytoskeleton and spine morphology and how, within our model, *pushing* the spine membrane at the location of the head effectively *pulls* the spine membrane inwards at the location of the spine-neck. Within our paradigm of the conservation of membrane, directly applying a contractile force *τ* at one or more locations along the spine-neck can achieve the same result. This is possible due to the nature of the spine membrane, which can be regarded as a two-dimensional fluid—that is, contracting the spine-neck effectively channels membrane to the spine-head. Thus, applying a line tension can aid in the transition from an immature to a mature spine with a long, thin neck and bulbous head. Possible candidates for such line tensions are anchoring molecules (such as the WASP/WAVE network [14, 33]) septin-complexes that form ring-like structures [34], spectrin [35] and recent reports of ring-like F-actin structures in spine-necks [36]. Next, we will show that anchoring molecules or ring-like complexes are able to apply sufficient contractile force along the spine-neck.

**Fig 4:**
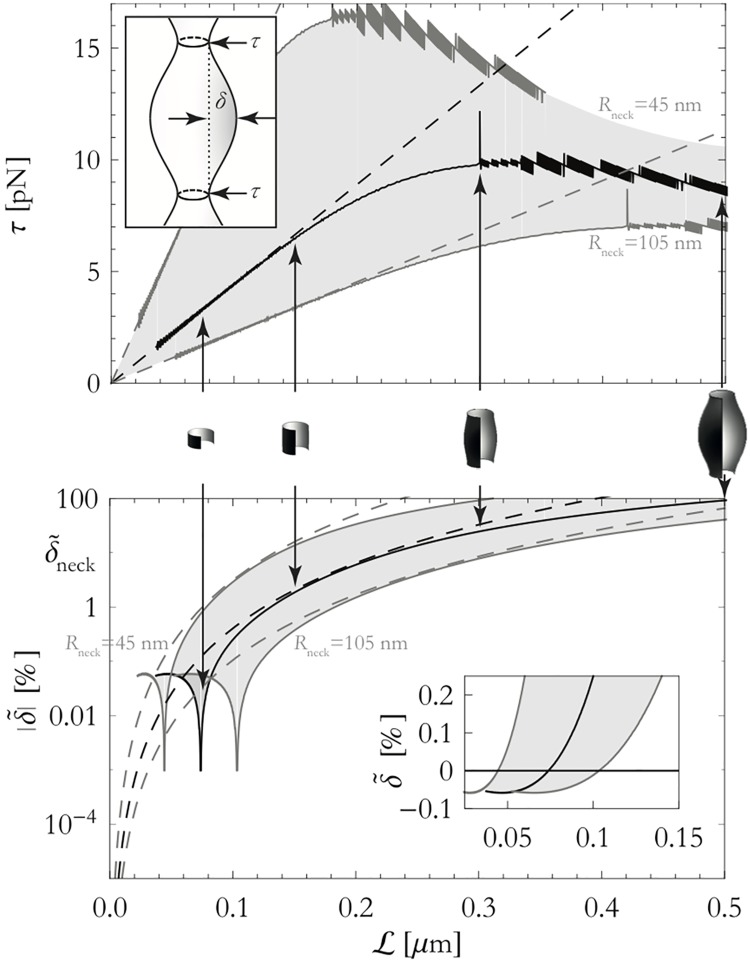
Overview of effect of constrictions on the shapes of spine-necks. Results of simulations that have been performed using the energy functional Eq (1) (solid curves) and theoretical model that treats these shapes as cylinders (dashed curves). The computations have been performed for *R*_neck_ = 45…105 nm as indicated in the figure. Black lines corresponds to *R*_neck_ = 75 nm. We used *K*_*b*_ = 5 × 10^−19^ *J* for these computations [11]. Top panel: The line tension *τ* as a function of the distance L between the line tensions. Inset shows how line tension *τ* and ‘unduloid amplitude’ *δ* are defined. Bottom panel: The absolute value of the reduced ‘unduloid amplitude’, $\left|\tilde{\delta }\right|$. Inset panel shows the reduced ‘unduloid amplitude’ where it crosses $\tilde{\delta }=0$. Indicated is ${\tilde{\delta }}_{\text{neck}}=10\%$, the approximate amplitude of variations in the width of the spine-neck, corresponding to *τ* ≈ 6.5 − 15pN.

A line tension can be included in our models by adding a term τC to the Canham-Helfrich free energy Eq (1), where *τ* is the line tension and $C=2\pi R_{\text{neck}}$ is the circumference of the spine-neck. The line tension can be measured thus τ=-∂E/∂C, where E is the bending energy of the shape. In Fig 4 it can be seen that the line tensions are typically in the order of piconewtons. As a consequence of one or a number of such line tensions we find ‘unduloidal’ spine-necks. Some representative shapes along with the required line tension have been reproduced in Fig 4. The shapes are characterized by an unduloid amplitude *δ* which describes the maximum deviation from the base value *R*_neck_ (we have chosen to use the relative unduloid amplitude $\tilde{\delta }=\delta /R_{\text{neck}}$).

Although we have not been able to find publications that measure the ‘unduloid amplitude’ $\tilde{\delta }$ for spine-necks, we have calculated this is in the order ∼10% or less (see S1 File for details). Using this value of $\tilde{\delta }$, we find that—if line tensions are responsible for the typical spine-morphology—the line tensions need to be placed at distances of L≈0.14-0.33μm, as can be readily verified by examining Fig 4. Then, computing the line tension (using numerical values *R*_neck_ and *K*_*b*_ from Table A in S1 File) corresponding to L≈0.14-0.33μm, we find that each of the line tensions experiences a load of *τ* ≈ 6.5 − 15pN. This, too, can be verified by examining Fig 4.

We are aware of various candidates for anchoring membrane to the cytoskeleton, such as L-selectin, *β*_2_ integrins and CD45. The literature reports that these three candidates have rupture forces respectively 25 − 45pN, 60 − 120pN and 35 − 85pN [37]. Even the lowest values of these three ranges is almost double our highest estimate for the required line tension. Therefore, it is safe to conclude that anchoring molecules can withstand the mechanical forces that are required in order to constrain the spine-neck to *R*_neck_ = 45 − 105 nm.

Since spine-necks typically have lengths of 0.2 − 2*μ*m (Table A in S1 File), we find that the number of line tensions that needs to be placed is 1 − 14, with a typical distance between the constrictions of L≈0.14-0.33μm. Ring-like septin-complexes that localize to dendritic spines do not have these properties *in vivo*, so that we can rule out septin-complexes as being solely responsible for constricting spine-necks (S1 File). In contrast, it has recently become evident that ring-like F-actin structures are consistently found across the entire length of spine-necks [36]. Indeed, the spacing between these ring-like structures is 194 ± 35nm [36], well within our predicted range. These F-actin rings are found *in addition* to vertically aligned F-actin filaments with rather consistent distances between them, thus suggesting a role for F-actin rings in stabilizing long, thin spine-necks.

## Conclusions

We study the physical mechanisms that determine the morphology of dendritic spines. In particular, we investigate the ability of the actin cytoskeleton to change the size and shape of spines. We find that the most striking primary features of spine growth and spine morphology can be straightforwardly understood as a consequence of the trade-off between the elastic properties of the spine membrane and the forces actively generated by the actin cytoskeleton. Specifically, we show that the initiation and formation of dendritic filopodia may be rationalized on the basis of the protrusive forces of the actin cytoskeleton. Using realistic estimates for the number of actin filaments involved, we find that the dimensions of the filopodia in our models agrees well with the observed dimensions of newly formed protrusions in the developing neuron.

We have also studied spine maturation, the process characterized by a morphological transition from a filopodium or thin spine to the mature mushroom-like spine. Using models based on the coupling between the actin cytoskeleton and the spine membrane, we find that the combined dynamics of branched actin and aligned actin inherently results in a mushroom-like morphology. Indeed, single-molecule studies show that the concentration of the actin nucleation factor Arp2/3 is greatly increased after spine stimulation [1, 38]. Moreover, proteins that align actin filaments, such as drebrin, *α*−actinin and CaMKII*β* [1] exhibit a substantial decrease in relative concentration concomitant with spine-head enlargement [38]. Interestingly, the protein fascin can also bundle actin filaments, and is indeed found in growth-cone filopodia, but not at the spine-neck [1, 5].

**Fig 5:**
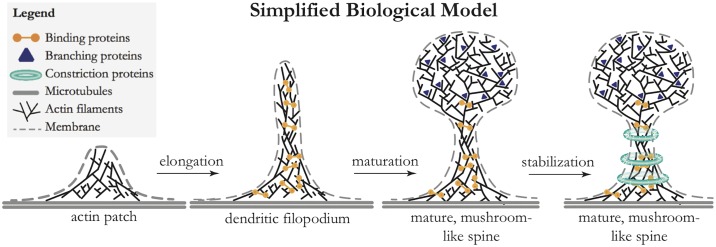
Cartoon of the proposed morphological stages in filopodium formation and spine maturation and their relation to various factors. We propose that *binding proteins*, such as drebrin, *α*−actinin and CaMKII*β* [1], may be involved in aligning the actin filaments away from the dendritic shaft. Branching proteins are necessary for effecting the transition to a branched actin organization and the large number of actin filaments in mature spine-heads. A candidate protein for this would be Arp2/3, a protein that localizes to the spine-head [1, 5]. Various proteins that constrict the spine-neck possibly aid in the stabilization of a long, thin spine-neck. Of these stabilizing factors, a strong candidate (that definitely localizes consistently to spine-necks) is the ring-like F-actin complex [36]; another candidate is the ring-like septin-complex [34, 39, 40].

Additionally, we have discussed how ring-like complexes and anchoring proteins may aid the stabilization of a long, thin spine-neck. In this regard, our models suggest a stabilizing role for ring-like F-actin structures that are consistently found in spine-necks, with concentrations closely matched by our predictions [36]. We condense our conclusions pertaining these actin factors in the form of a cartoon, Fig 5, displaying the importance of branching proteins to effect the transition in actin organization and the possible stabilization factors such as septins and ring-like F-actin complexes.

Within our models, predictions for various morphological quantities, such as the neck radius *R*_neck_, the ratios *R*_head_/*R*_neck_ and $V_{\text{head}}/V_{\text{neck}}$ and the typical number of ring-like stabilizing factors compare well with experimental data [3, 13, 36]. This agreement highlights the applicability of our simple model based on a competition between the forces generated by membrane deformation and those generated by actin organization and stabilizing factors. Summarizing, the suggested roles of branched and aligned actin organizations, combined with ring-like stabilizing factors, suggest novel experiments analyzing (possibly, even, altering) the localization of such proteins in dendritic spines.

## Supporting information

S1 File Contains supplementary information w.r.t. our model parameters, an estimate for the typical number of actin filaments in spine-heads, an estimate for the standard deviation in the width of spine-necks, a comparison of the ensemble of constant surface-area and constant surface-tension and a more detailed description of the shape equations that we have used. (PDF)
