# Supplementary material for: Biophysical model of the role of actin remodeling on dendritic spine morphology
Source: PLoS One. 2017 Feb 3;12(2):e0170113. doi: 10.1371/journal.pone.0170113 (PMC5291493; doi:10.1371/journal.pone.0170113)
Supplement: S1 File — (PDF) [file pone.0170113.s001.pdf]

Actin Remodeling and Polymerization Forces Control Dendritic Spine Morphology

C.A. (Karsten) Miermans*[1], R.P.T. (Remy) Kusters[1], C.C. (Casper) Hoogenraad[3], and C. (Cornelis) Storm[1,2]

[1] *Theory of Polymers and Soft Matter, Department of Applied Physics, Eindhoven University of Technology, The Netherlands*
[2] *Institute for Complex Molecular Systems, Eindhoven University of Technology, The Netherlands*
[3] *Cell Biology, Faculty of Science, Utrecht University, The Netherlands*

January 26, 2017

Supporting Information

Table A. Various 'ball-park' figures of dendritic spines that we use as model parameters and figures for comparison.

**Code**	**Quantity**	**Typical Scale**	**Source**
N	Number of actin filaments in spine-head (see "Estimate for the Number of Actin Filaments", S1 File)	~ 71	(1)
R_{base}	Radius of the base of the spine (viz. where the spine is connected to the dendritic membrane). This quantity was estimated on the basis of microscopy images published by (2).	$\sim 300\,\text{nm}$	(2)
R_{neck}	Radius of a typical spine-neck	75 ± 30 nm	(3)
R_{head}	Radius of a typical spine-head	220 ± 154 nm	(3)
$\mathcal{L}_{\text{neck}}$	Length of a typical spine-neck	$0.2 - 2$ μm	(2)
$\mathcal{L}_{\text{filop.}}$	Length of a typical filopodium	$0.9 - 10\,\mu$m (4) (mean $\approx 5\,\mu$m (5))	(4, 5)
ℓ	Length that actin filament extends upon one polymerization step	2.2 nm	(6)
$\mathcal{A}_{\text{neck}}$	Surface-area of a typical spine-neck	0.24 ± 0.17 μm^2	(3)
$\mathcal{A}_{\text{head}}$	Surface-area of a typical spine-head	0.61 ± 0.57 μm^2	(3)
$\mathcal{A}_{\text{filop.}}$	Surface-area of a typical filopodium (this was calculated using $\mathcal{A}_{\text{filop.}} \approx 2\pi R_{\text{filop.}} \mathcal{L}_{\text{filop.}}$ with $R_{\text{filop.}}, \mathcal{L}_{\text{filop.}}$ from (4, 5))	$0.85 - 16\,\mu\text{m}^2$ (mean $\approx 6.3\,\mu\text{m}^2$)	(4, 5)
f_{actin}	Average actin polymerization force	3.8 pN	(6)
K_b	Bending rigidity of lipid bilayer membrane	5×10^{-19} J	(7)

*Corresponding author, c.miermans@lmu.de

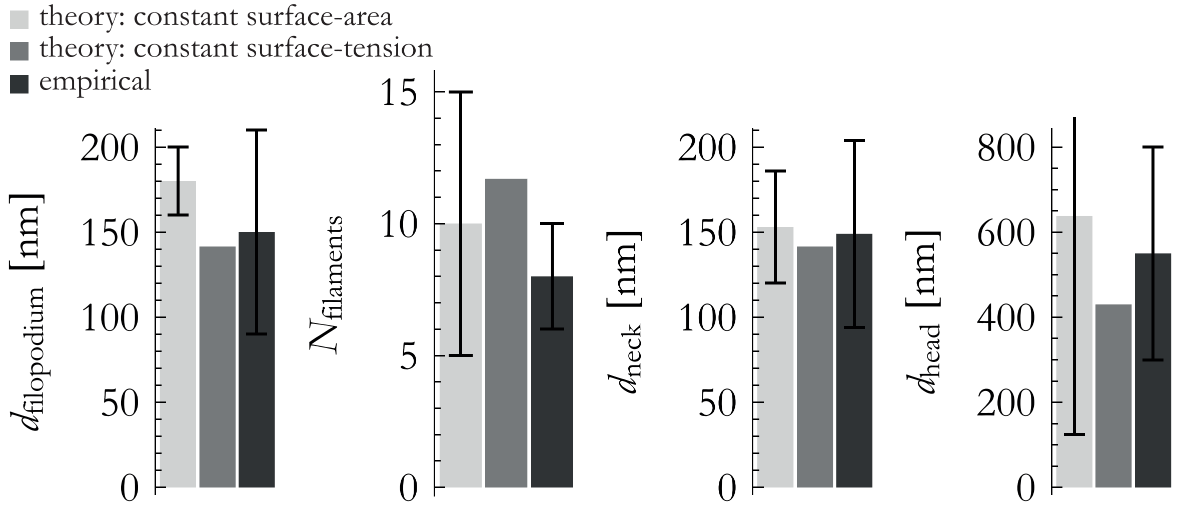

Figure 1: Comparison of the ensemble of constant surface-area versus the ensemble of constant surface-tension with experiments. Figures of comparison were: The dendritic filopodium thickness $d_{\text{filopodium}}$, the number of filaments observed inside dendritic filopodia N, the neck-thickness of mature spines d_{neck} and the head-width of mature spines d_{head}. Comparison of the predictions with experiment shows that the models in both ensembles predict the correct order of magnitude for all morphological characteristics, although significant differences are observed between the two ensembles. Experimental data on widths was taken from (2), number of filaments $N_{\text{filaments}}$ required to produce dendritic filopodia was estimated from EM images published in (1). Whiskers denote maximum and minimum values of the corresponding data. Note that the standard deviation in measured quantities is necessarily lower, and often much lower, than these lower and upper bounds.

0.1 Septin Complexes are Unlikely Required for Effecting the Transition to Mature Spines

Although septin-complexes are found consistently along spine-necks (8–10), they are only reported to be positioned at the *base* of the spine and *not* along the full length of the spine-neck. Our models predict that it is required to place line tensions along the full length of the spine-neck in order to constrain it, and therefore we can refute septin-complexes as being solely responsible for constraining the long, thin spine-necks. Moreover, the assembly of septins into ring-like structures has an associated time-scale in the order of minutes (11). Hence, we find that cytoskeletal remodeling—which can performed on the time-scale of fractions of a second (6)—is much more rapid than positioning these septins. From these observations combined, we hypothesize that ring-like septin-complexes or anchoring proteins are not required for the transition from filopodium to mature spine, but could plausibly aid in the stabilization of these mature, mushroom-like spines.

0.2 Estimate for the Number of Actin Filaments

We counted the number of actin filaments as 20 on $\sim 20\%$ of the surface-area resulting in ~ 100 filaments for one entire spine-head as published by (1). Then, noting that *on the average* the filaments

are not oriented perpendicular to the membrane–but rather at an angle $\pi/4$, we find the *effective* number of actin filaments to be $\sim 100 \cdot \cos(\pi/4) \approx 71$. This number falls within the range for the number of polymerizing filaments $N = 50 - 150$ we derived from data published in (12) ((12) has published the density of non-stationary actin molecules, which we integrated over the surface area to obtain a measure for the number of polymerizing filaments).

0.3 Standard Deviation in Spine-Neck Width

We measured the width of the spine-neck of images by (2) by fitting the intensity of the profile with Gaussian distributions along the axis of the spine-neck. We asserted that the standard deviation of these Gaussians is a measure for the width of the spine-neck. Then, we computed the relative variation in these widths. Using this method, the relative variation in the width of the spine-neck was found to be 13.5%.

0.4 Shape Equations

Taking the first variation of the Canham-Helfrich energy functional, and insisting that the first variation $\delta\mathcal{E}$ is zero under all possible infinitesimal perturbations results in a differential equation that describes stationary shapes $\{r(s), \psi(s)\}$. The stationary shapes include shapes corresponding to an energetic minimum, an energetic maximum or a saddle point in the energy functional. A seminal paper by (13) describes the higher-order variations, from which we can infer the class of stationary point. We will not discuss this technical difficulty in this publication, although we have used numerical perturbative methods to determine which shapes correspond to energetic minima. This differential equation, that we shall henceforth call the *shape equation*, is (14, 15)

$$\psi^{(3)} = -\frac{1}{2}(\psi')^3 - \frac{2\cos\psi}{r}\psi'' + \frac{3\sin\psi}{2r}(\psi')^2 + \frac{\bar{\sigma}}{r}\sin\psi + \frac{3(\cos\psi)^2 - 1}{2r^2}\psi' + \bar{\sigma}\psi' - \frac{(\cos\psi)^2 + 1}{2r^3}\sin\psi - \bar{p}, \quad (1)$$

where we have dropped the s–dependencies and $\bar{\sigma} \equiv \sigma/K_b$. Most publications that we have consulted make reference to second-order shape equations (14), but–in accordance with (15)–we find the third-order shape equation 1 to be numerically substantially more stable. The second-order shape equations, e.g. found by taking the first integral of 1, is used to find boundary conditions for ψ''. This equation is (15)

$$\psi'' \cos\psi = -\frac{1}{2}(\psi')^2 \sin\psi - \frac{(\cos\psi)^2}{r}\psi' + \frac{(\cos\psi)^2 + 1}{2r^2}\sin\psi + \bar{\sigma}\sin\psi - \frac{1}{2}\bar{p}r - \frac{\bar{f}}{r}, \quad (2)$$

where $\bar{f} \equiv f/2\pi K_b$. Although the point force f does not show up in the shape equation 1, it does enter in the determination of the correct boundary conditions through equation 2.

In this paper, we have used the ensemble of a prescribed surface-area available to the shape (for more details and rationale, see the main text). We have used its conjugate variable, the surface-tension σ, with a shooting-and-matching technique to constrain the surface-area to a given value. Similarly, we use respectively the vertical force f, the initial curvature $\psi'(0)$ and the total arc-length $\mathcal{S}$ as Lagrange multipliers (and hence, shooting variables) to generate shapes with a prescribed height $\mathcal{L}$, total surface-area $\mathcal{A}$ and to match them to the $z-$axis (i.e. $r(\mathcal{S}) = 0$). For more information on this numerical technique, we refer to (16)).

References

1. Korobova F, Svitkina TM. Molecular Architecture of Synaptic Actin Cytoskeleton in Hippocampal Neurons Reveals a Mechanism of Dendritic Spine Morphogenesis. Molecular Biology of the Cell. 2010;21(22):4042–4056. doi:10.1091/mbc.E09.

2. Tonnesen J, Katona G, Rózsa B, Nägerl UV. Spine neck plasticity regulates compartmentalization of synapses. Nature Neuroscience. 2014;17(5):678–85. doi:10.1038/nn.3682.

3. Harris KM, Stevens JK. Dendritic Spines of CA1 Pyramidal Cells in the Rat Hippocampus : Serial Electron Microscopy with Reference to Their Biophysical Characteristics. Journal of Neuroscience. 1989;9(8):2982–2997.

4. Evers JF, Muench D, Duch C. Developmental relocation of presynaptic terminals along distinct types of dendritic filopodia. Developmental Biology. 2006;297(1):214–227. doi:10.1016/j.ydbio.2006.05.012.

5. Ziv NE, Smith SJ. Evidence for a Role of Dendritic Filopodia in Synaptogenesis and Spine Formation. Neuron. 1996;17(1):91–102. doi:10.1016/S0896-6273(00)80283-4.

6. Mogilner A, Oster G. Force generation by actin polymerization II: the elastic ratchet and tethered filaments. Biophysical Journal. 2003;84(3):1591–1605. doi:10.1016/S0006-3495(03)74969-8.

7. Semrau S, Idema T, Holtzer L, Schmidt T, Storm C. Accurate Determination of Elastic Parameters for Multicomponent Membranes. Physical Review Letters. 2008;100(8):088101. doi:10.1103/PhysRevLett.100.088101.

8. Ewers H, Tada T, Petersen JD, Racz B, Sheng M, Choquet D. A Septin-Dependent Diffusion Barrier at Dendritic Spine Necks. PloS one. 2014;9(12):e113916. doi:10.1371/journal.pone.0113916.

9. Mostowy S, Cossart P. Septins: the fourth component of the cytoskeleton. Nature Reviews Molecular Cell Biology. 2012;13. doi:10.1038/nrm3284.

10. Tada T, Simonetta A, Batterton M, Kinoshita M, Edbauer D, Sheng M. Role of Septin Cytoskeleton in Spine Morphogenesis and Dendrite Development in Neurons. Current Biology. 2007;17(20):1752–1758. doi:10.1016/j.cub.2007.09.039.

11. Kinoshita M, Field CM, Coughlin ML, Straight AF, Mitchison TJ. Self- and Actin-Templated Assembly of Mammalian Septins. Developmental Cell. 2002;3(6):791–802. doi:10.1016/S1534-5807(02)00366-0.

12. Frost NA, Shroff H, Kong H, Betzig E, Blanpied TA. Single-molecule discrimination of discrete perisynaptic and distributed sites of actin filament assembly within dendritic spines. Neuron. 2010;67(1):86–99. doi:10.1016/j.neuron.2010.05.026.

13. Zhong-Can OY, Helfrich W. Bending energy of vesicle membranes: General expressions for the first, second, and third variation of the shape energy and applications to spheres and cylinders. Physical Review A. 1989;39(10):5280–5288. doi:10.1103/PhysRevA.39.5280.

14. Jülicher F, Seifert U. Shape equations for axisymmetric vesicles: A clarification. Physical Review E. 1994;49(5):4728–4731. doi:10.1103/PhysRevE.49.4728.

15. Derényi I, Jülicher F, Prost J. Formation and Interaction of Membrane Tubes. Physical Review Letters. 2002;88(23):238101. doi:10.1103/PhysRevLett.88.238101.

16. Heath MT. Scientific Computing: An Introductory Survey. McGraw-Hill; 2002.
